# Co-creating an Inclusion, Diversity, Equity, and Accessibility (IDEA) Strategy in a Pan-Canadian Health Data Research Network

**DOI:** 10.23889/ijpds.v11i1.3429

**Published:** 2026-08-13

**Authors:** Morgan Stirling, Laura Bowler, Jeffrey Morgan, David Yang, Lisa Nowlan, Kate Millberry, Kelli Buckreus, Jennifer Hagen, Jannath Naveed, Kim McGrail, Nathan Nickel, Amy Freier

**Affiliations:** 1 University of Manitoba, Winnipeg, MB, Canada; 2 Health Data Research Network Canada; 3 Manitoba Centre for Health Policy, Winnipeg, MB, Canada; 4 University of British Columbia, Vancouver, BC, Canada; 5 Canadian Institute for Health Information, Canada; 6 Alberta SPOR SUPPORT Unit, Calgary, AB, Canada; 7 University of Calgary, AB, Canada; 8 DataNB, Fredericton, NB, Canada; 9 University of New Brunswick, Fredericton, NB, Canada; 10 Memorial University of Newfoundland, St. John's, NL, Canada; 11 Simon Fraser University, Burnaby, BC, Canada

**Keywords:** IDEA, inclusion, equity, diversity, accessibility, administrative data, data equity, Health Data Research Network Canada, Strategy, strategic planning

## Abstract

Inclusion, Diversity, Equity, and Accessibility (IDEA) are increasingly recognised as essential to advancing population health research and addressing structural inequities. Yet, few publications describe how to develop IDEA strategies, leaving organisations with limited guidance on replicable processes. Here, Health Data Research Network Canada (HDRN Canada) details the steps it took to establish its own IDEA Strategy.

The strategy was developed through an iterative five-phase process. Key steps included creating a project charter, defining shared governance and consensus-based decision making, and using professional facilitation to foster broad participation across member organisations. Visible executive sponsorship was critical to strategy development and implementation.

The resulting strategy identifies four interconnected action areas: Learning and Unlearning, Facilitating IDEA in Research, Cultivating Trust and Reciprocity, and Providing Leadership and Advocacy to embed IDEA in organisational operations and research practices. HDRN Canada’s experience demonstrates how a national distributed research network of organisations that work together to support multi-jurisdictional research can use best practices to transform IDEA principles into a concrete, actionable framework. This work offers a transferable model for population health research organisations seeking to integrate IDEA within their organisations and across the research ecosystem.

## Introduction

### Background

Inclusion, Diversity, Equity, and Accessibility (IDEA) are increasingly recognised as foundational principles for advancing health research and addressing structural inequities. A proliferation of resources and best practices reflects a growing movement to integrate IDEA across research environments [1–4]. IDEA-related strategic planning can help organisations interpret best practices and IDEA concepts in the context of a particular field or domain and articulate tailored approaches the organisation will take to promote equitable, accessible, and inclusive research. However, there is limited published detail describing how organisations develop IDEA strategies and translate broad principles into contextually relevant priorities and actions [5–7]. This lack of practical guidance limits opportunities for other organisations to learn from and adapt established approaches, particularly in specialised research settings such as population data science.

As the science of data about people, population data science has long emphasised the responsible use of data for public good and the ethical challenges associated with handling sensitive data [8]. At the same time, there is growing recognition that data, systems, and research practices underpinning this field can reproduce systemic biases and inequities and contribute to harm among systematically marginalised populations [9–12]. Addressing these challenges requires more than technical improvements. It also necessitates examining how power operates within research structures and processes, including who defines research priorities, how expertise is valued, and the extent to which communities are involved in governance and decision making [11, 13]. This imperative is especially salient in multi-jurisdictional research environments, such as distributed research networks, where researchers and research support organisations must navigate complex administrative processes, diverse data systems, varying data governance requirements, and differing expectations regarding public and community involvement [9, 10, 14–16].

Health Data Research Network Canada (HDRN Canada) is a not-for-profit corporation consisting of a pan-Canadian distributed network of organisations collectively focused on supporting use of population-level data to advance health and health equity. Established in 2019 with 18 member organisations, HDRN Canada is a distributed network where most member organisations hold linkable population health and health-related data or have mandates relating to the access or use of those data. HDRN Canada is organised into several cross-cutting Teams that support the network’s strategic priorities, including initiatives related to data access, public engagement, Indigenous data governance, training and capacity building, and equity-oriented data practices. Network members share knowledge and identify opportunities to collaborate and support researchers conducting multi-regional research, share and build advanced analytics, expand types and sources of data linkage, create infrastructures to improve access to and collection of data, and establish partnerships with relevant parties, Indigenous communities, and other knowledge users [17]. In this context, advancing Inclusion, Diversity, Equity, and Accessibility (IDEA) is closely connected to, while distinct from, commitments to the principles of OCAP® and broader Indigenous Data Sovereignty, which establish specific rights and responsibilities related to the governance and use of Indigenous data.

In its 2022–2026 Strategic Plan, HDRN Canada formally included a strategic objective related to IDEA and committed to developing a comprehensive IDEA strategy and action plan that would embed IDEA across the organisation’s five strategic goals [18]. Although many publications describe why IDEA is important and identify promising practices, few provide detailed accounts of how organisations can develop tailored strategies in complex research environments. To address this gap, this paper describes the structured, participatory process HDRN Canada followed to co-create its IDEA Strategy. Our approach was informed by principles of co-creation and participatory strategic planning, which emphasize engaging diverse stakeholders throughout the planning process to build shared ownership and ensure that resulting priorities reflect the needs and perspectives of those involved [19–21]. By focusing on strategy development itself rather than implementation, this paper offers a practical roadmap to other organisations seeking to translate IDEA principles into contextually relevant priorities and actions within population data science.

### Why IDEA in Population-based Data Research?

Population-based data research using administrative data has significant potential to improve population health outcomes. However, the systems and practices underpinning this field can also reproduce inequities and contribute to harm [22, 23]. Some of these harms are related to the ways different populations are represented and described in research. For example, administrative data sets used in population data research often rely on binary or inconsistently defined sex and gender variables, limiting researchers’ ability to accurately identify and describe transgender, non-binary, and gender diverse populations [24]. Inadequate gender measures also increase the risk of research using outdated or stigmatizing terminology when describing this community. Similar challenges exist when data are linked across systems that collect demographic information in different ways or when important variables such as race and ethnicity are unavailable or inconsistently captured [25, 26].

Other harms arise through the invisibility of populations within data systems and research. The absence of sociodemographic indicators related to social exclusion, such as housing status, substance use disorders, engagement in legal sex work, and incarceration experiences, makes it difficult to identify and address the health needs of groups experiencing marginalization [27]. Individuals may also be missing from administrative data altogether because of barriers to service access, stigma, or concerns about safety [28]. As a result, the benefits of population health research may not be equitably distributed, and groups that are underrepresented in data may be excluded from decisions regarding health and social service planning and resource allocation [29–31].

There is also increasing reliance on artificial intelligence and machine learning methods in population health research. While these approaches may improve efficiency and analytic capacity, they are susceptible to biases arising from missing data, historical inequities, and assumptions embedded in model development [32]. Studies have identified algorithmic biases, including the omission of social context and reliance on potentially biased clinical decisions, that may reinforce rather than reduce inequities [33]. Similar challenges arise when researchers develop computational phenotypes to identify specific populations in large datasets. These methods require defining inclusion criteria that are shaped by both human assumptions and data limitations. For example, Rich et al. developed a computational phenotype to identify transgender individuals using diagnostic codes and exogenous sex hormone prescriptions [34]. Although innovative, this approach may inadvertently reinforce the assumption that all transgender people undergo some form of medical transition.

Data used in population health research are typically collected by governments, healthcare organisations, and other institutions for administrative rather than research purposes [35]. Because researchers are often removed from the original data collection context, they may overlook important historical, social, and structural factors that shape how data are generated and interpreted [13, 36, 37]. This can lead to analyses that decontextualize communities and perpetuate harmful narratives. For example, interpreting the overrepresentation of Indigenous peoples in justice or child welfare systems without acknowledging the impacts of colonialism, racism, and intergenerational trauma can reinforce deficit-based stereotypes [38, 39].

Together, the absence of meaningful sociodemographic data, the invisibility of marginalised populations, and research practices that overlook structural context can contribute to epistemic harm by privileging dominant ways of knowing while dismissing community knowledge and lived experience [13, 40]. These practices shape assumptions about who is visible, who counts, and whose experiences are considered legitimate within research, ultimately limiting the ability of population health research to advance health equity [41].

There is growing recognition of these harms and ongoing efforts to address them. Recommendations include improving sociodemographic data collection, engaging communities affected by research, diversifying evaluation methods, and critically examining how systems of oppression and intersectionality shape both data and research practices [16, 42–44]. HDRN Canada’s IDEA Strategy responds to this imperative by providing a pathway to embed IDEA considerations within research practices and across organisational structures.

## Methods Used to Develop the Strategy

### How the Strategy was Developed

**Figure 1:**
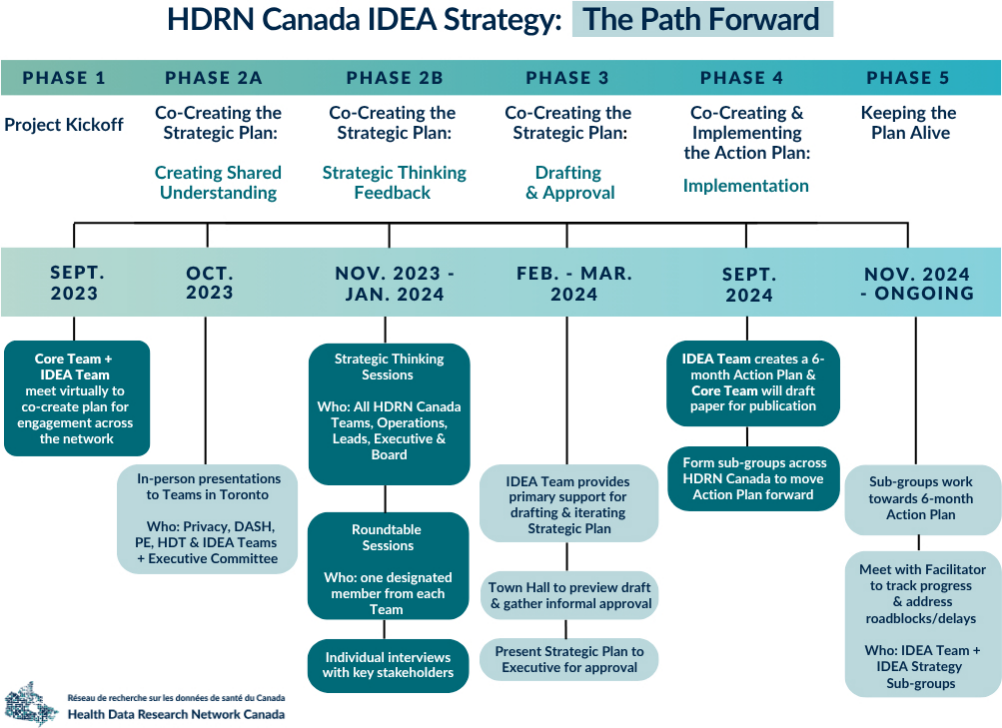
IDEA Strategy Development Process

HDRN Canada created its IDEA Strategy through a structured five-phase, organisation-wide process designed to support shared leadership, collaborative planning, and inclusive engagement (Figure 1). The process began with preparatory work to assess organisational readiness and identify priorities, followed by activities to establish governance structures, create a shared understanding of the strategy’s purpose and approach, engage stakeholders in strategic thinking and feedback, and iteratively draft and refine the strategy. Although implementation activities are underway, the focus of this paper is on the process used to co-create the strategy. The implementation component shown in Figure 1 refers to the strategic directions described in the following section rather than an additional phase of work.

#### Phase 0: Pre-planning and Setting the Course

As part of its initial IDEA-related work, HDRN Canada conducted an environmental scan across member organisations to identify: how they respectively organise and support IDEA activities, how resources were allocated towards this work, and which IDEA-related priorities were most important within their organisation. A detailed description of the environmental scan methods and findings has been published elsewhere [45]. In brief, the scan revealed that while many organisations across the network were actively advancing IDEA, there were substantial differences in funding, staffing, and expertise across member organisations. These findings highlighted an important role for HDRN Canada in connecting emerging IDEA concepts to population data science, developing shared guidance and resources, amplifying the work of member organisations, and helping address areas where local capacity was limited.

#### Phase 1: Project Kickoff (September 2023)

The environmental scan and an interim evaluation of the network as a whole provided a strong rationale for HDRN Canada’s leadership to allocate dedicated financial and human resources to support development of the IDEA Strategy. This feedback also highlighted the importance of visible leadership in communicating the organisation’s commitment to IDEA and establishing expectations for broad participation across the network.

In addition to securing support and resources, HDRN Canada established a Project Team consisting of three distinct groups with complementary roles in guiding and supporting strategy development:

1. **Core Team**: A small leadership group responsible for project management and for overseeing development and implementation of the IDEA Strategy.
2. **IDEA Team**: The Core Team plus representatives from member organisations and HDRN Canada Teams who contributed to and co-created the strategy.
3. **Facilitation Team**: The Core Team and an external facilitator responsible for coordinating and delivering engagement activities.

HDRN Canada recognised that sufficient expertise in IDEA and population health research already existed across the network. As a result, an external facilitator was engaged primarily for expertise in group process design and facilitation rather than for subject matter expertise.

#### Phase 2A: Co-creating the Strategic Plan - Creating Shared Understanding (October 2023)

Once commitments were secured and the Project Team established, HDRN Canada moved to structure the IDEA Strategy development process formally. This required setting shared expectations, roles, and ground rules to guide the collaborative work ahead.

As a distributed network with member organisations spanning a wide geographical region and multiple time zones, we employed a variety of flexible approaches to engage with one another. This was accomplished using online meetings and asynchronous communication (i.e., email and Mural Board). With guidance of the external facilitator, the Project Team co-created a charter that defined project objectives, identified scope, and articulated particular preferences for co-creation and consensus-based decision making. In addition, the Project Team developed a Strategic Planning Context Document that complemented the project charter by providing a concise overview of the rationale for the strategy, its intended goals and scope, roles and expectations, and the participatory approach that would guide its development. Unlike the charter, which established working agreements within the Project Team, this document was shared broadly across the network to support engagement activities and ensure participants had a common understanding of the purpose and process of strategy development. It also served as a reference point throughout the project.

#### Phase 2B: Co-creating the Strategic Plan - Strategic Thinking Feedback (November 2023–January 2024)

Co-creation activities were guided by the Technology of Participation (ToP). This process involves a collection of structured facilitation methods that enable groups to think and plan together [46]. Developed by the Institute of Cultural Affairs, ToP is a process that supports participation transforming into long-term commitment and follow-through [47]. The external facilitator was ToP certified. At the outset, the Facilitation Team met with each HDRN Canada Team to review the process and describe the Strategic Planning Context Document. These preparatory steps ensured that participants, regardless of prior familiarity with IDEA, had the context needed to contribute.

The engagement process unfolded through three key activities:

- **Strategic Thinking Sessions:** Facilitated sessions were conducted with each HDRN Canada Team separately using virtual whiteboards (Mural). Participants reflected on HDRN Canada’s IDEA journey, articulated a collective vision for 2026, identified systemic contradictions that could impede progress, and generated strategies to address these challenges.
- **Key Informant Interviews:** The facilitator conducted six one-on-one interviews with individuals from across the network. These interviews helped surface perspectives that might not emerge in group settings and ensured that the strategy reflected a broad range of experiences.
- **Roundtable Discussions:** After insights from the strategic thinking sessions and interviews were synthesised into thematic clusters, representatives from across the network reconvened in facilitated roundtables to reach consensus on the strategy’s vision, priorities, and strategic directions.

During this phase, participants were able to contribute both synchronously during live sessions and asynchronously through shared digital tools. This flexible and participatory approach fostered broad engagement and a strong sense of shared ownership and accountability across the network.

#### Phase 3: Co-creating the Strategic Plan – Drafting and Approval (February–March 2024)

Once outputs from the Strategic Thinking Sessions, Key Informant Interviews, and Roundtable Discussions, including participants’ reflections on HDRN Canada’s IDEA journey, collective vision, systemic contradictions, and potential strategies for change, were compiled, a sub-group was established from the Project Team to draft the initial strategy. This group was responsible for consolidating and theming participants’ contributions to develop a working draft. This working draft was translated into both English and French and distributed across HDRN Canada. Following dissemination, we initiated a multi-pronged feedback process across the network.

1. **Surveys** were distributed to each HDRN Canada Team to assess alignment with HDRN Canada’s strategic priorities, identify which proposed strategies were most relevant to their work, and surface any implementation concerns.
2. **Facilitated feedback sessions** were held with each HDRN Canada Team, the Public Advisory Council, and organisational leaders to member-check the draft and refine its content.
3. **A network-wide town hall** provided an opportunity for participants to reflect on whether the proposed strategy aligned with their expectations and with HDRN Canada’s broader goals.

After incorporating feedback, the revised strategy was presented to HDRN Canada’s Executive Committee and Board of Directors. These bodies reviewed the document, provided final input, and formally endorsed the strategy. Their approval signalled a strong organisational commitment to the strategy’s vision, principles, and actions.

#### Phase 4: Co-creating & Implementing the Action Plan – Implementation (April 2024)

Following approval of the IDEA Strategy, sub-groups were established to begin translating each strategic priority into actionable initiatives. Each sub-group was led by members of the Project Team, with additional participants identified from across the network based on their interest and relevant expertise.

During a series of meetings, each sub-group discussed potential initiatives and used consensus-based decision making to identify initial activities that could support progress toward each strategic priority. Although implementation and evaluation are beyond the scope of this paper, these early planning activities informed the initial actions described in the following section and established a foundation for ongoing implementation across HDRN Canada. These actions remain ongoing.

### HDRN Canada's IDEA Strategy: Four Strategic Priorities for Action

As part of the co-creation process, participants identified four interrelated strategic priorities that together provide a framework for embedding IDEA across HDRN Canada’s governance, operations, and research infrastructure. These priorities reflect areas where participants believed coordinated action was needed to strengthen equitable data practices, build organisational capacity, and influence the broader population health research ecosystem.

#### Learning and Unlearning

As a largely academic-based data research support organisation, it is imperative HDRN Canada stays abreast of developments that connect IDEA to population health research. There has been a proliferation of work that makes this connection necessary, including data equity and its cognate terms: data justice, quantitative critical race theory, racialisation of data, causal fairness, and algorithmic justice [1–4]. Additionally, developments related to learning and unlearning about topics such as white supremacy, institutional and systemic bias, and cultural safety are also important, as they impact practices such as hiring, policies, the research environment, as well as access and data collection within health care.

With respect to the IDEA strategy development, the vast majority of participants expressed a need and desire to learn more about IDEA and its application to work within HDRN Canada and their member organisation. They stated the need for appropriate resources and dedicated time for learning, both at the individual and organisational levels. In this way, the Learning and Unlearning strategy compels HDRN Canada to remain up-to-date with best practices while also prioritising the time it takes to learn, internalise, and integrate IDEA in the population research space.

Initial activities under this strategy include internal and external priorities. Internally, there is ongoing development of IDEA competencies that articulate core, stretch, and specialised role-specific expectations for individuals working across the network. These competencies are intended to reinforce that everyone has a role to play in IDEA, guide continuing learning, and support the integration of IDEA into organisational and research practices. Externally, HDRN has established a public facing webinar series, *Big IDEAs About Health Data*, that showcases novel uses of disaggregated data as well as advancement in data research methods that embed inclusion, diversity, equity, and accessibility.

#### Facilitating IDEA in Research

As outlined earlier, gaps in data collection that affect the visibility of populations, limited knowledge about how data was collected, and the highly technical nature of data systems may present barriers to acknowledging and integrating equity in data processes and research [27, 33, 36]. Several frameworks have been developed to help address equity in data environments, such as the Data Equity Framework (We All Count) [48], the Toolkit for Centering Racial Equity Throughout Data Integration (Actionable Intelligence for Social Policy) [28], and the Disaggregated Demographic Data Collection Report (B.C. Human Rights Commission) [16].

During strategy development, participants described the need to refine recommendations from these resources so they could be applied within a Canadian administrative data landscape and shared with researchers. In addition, network members wanted to ensure that the data infrastructure tools that are developed also reflect leading practices, pushing the boundaries when it comes to the inclusion of contextual information about data that may inform equitable use. Participants agreed that HDRN Canada is well-positioned to support, advocate for, and bolster efforts to facilitate IDEA into existing and novel data use approaches and practices across the population data research ecosystem.

Under this priority, initial activities include the development of internal facing briefing notes examining the implications of IDEA for administrative data research and research infrastructure with a particular focus on common data models, concept dictionaries, phenotype libraries, and metadata. This ongoing work is helping to identify practical approaches for translating emerging data equity frameworks into guidance that can support researchers and member organisations.

#### Cultivating Trust and Reciprocity

Patient and public engagement has been one longstanding way of centring equity in research [49, 50]. However, questions remain about how best to involve patients and the public in multi-regional administrative data research. Disaggregated data presents the best opportunities for advocacy and policy change when used in a localised community [51]. When scaled for use at the national level, targeted interventions that would be best identified by patients or members of the public may get lost. Additionally, when doing multi-regional research, the question becomes which community or communities the researcher should build relationships and partner with. Limited funds and time, both hallmarks of academic research, become magnified when engagement is considered across communities, regions, and interests.

Participants in the Strategic Thinking Sessions, Interviews, and Roundtable Discussions expressed the need to find ways to scale engagement ethically and relationally, considering technical environment and regional differences. Key to this work is improving relationships with communities across Canada who have experienced or are at risk of experiencing data harms and ensuring purposeful and ongoing public and community involvement. As with all strong relationships, the importance of reciprocity was raised and echoed throughout discussion sessions.

Initial activities in this area include the development of a coordinated process guide to consider both IDEA and public engagement in HDRN Canada’s internal projects and initiatives. A grey literature scan, an internal survey, and engagement with HDRN Canada’s Public Advisory Council has informed the process guide. This work remains ongoing and is intended to promote more consistent, reciprocal, and thoughtful approaches to relationship building and community involvement across the organisation. While this initial step is focused internally, a subsequent action will be to extend the developed process guide for use by researchers in project planning.

#### Providing Leadership and Advocacy

Leadership is critical for embedding IDEA in an organisation’s culture, strategy, and operations. IDEA leaders must have knowledge, use knowledge, and cede power where needed. Academic leaders are especially good at knowledge accumulation and use; however, ceding power within bureaucratic academic and data research environments, which are often enshrined in colonial government structures of health, education, and social services remains challenging. Evidence shows that IDEA initiatives are often the first to be cut during economic hardship, yet this runs counter to the fact that systematically marginalised communities are hit hardest and earliest [52–54].

Throughout strategy development sessions, it was clear that HDRN Canada would require strong leadership not only to sponsor IDEA through appropriate change management channels, but also to provide resources and stay the course during difficult and uncharted conversations. Participants recognised that successful implementation of the framework elements described above requires strong, clear leadership from HDRN Canada’s Executive Committee, which is responsible for providing direction, and from HDRN Canada Team Leads, whose subject matter expertise can assist HDRN Canada to identify and incorporate IDEA. Moreover, in the spirit of reciprocity, participants saw it as HDRN Canada’s responsibility to openly share tools and resources developed in all other strategies as a way to demonstrate broader leadership and make change within the population health data research ecosystem as a whole.

Initial activities under this priority included the public release of the IDEA Strategy to share HDRN Canada’s approach with member organisations and the broader population data science community. Through 2025 a comprehensive list of actions was generated that would help improve IDEA in the population data science ecosystem, with a focus on activities that would have a large impact but that would need to be addressed with other data research organisations. After internal work to prioritise these activities, leadership in governance changes as they relate to IDEA was identified as a key challenge. Since that time, there has been a focus on evolving the internationally recognised Five Safes framework to better reflect IDEA, Indigenous Data Sovereignty, and Public Engagment. Work in this domain is expected to be released in 2027.

## Discussion

HDRN Canada’s experience demonstrates how a distributed research network can co-create an actionable IDEA Strategy while navigating the complexities of organisational change. The process aligns with best practices in co-creation and strategic planning, particularly with respect to engaging diverse stakeholders early and throughout the planning process, establishing transparent governance and decision making structures, creating psychologically safe and reflexive spaces for feedback, and building accountability mechanisms [19–21]. Three lessons were especially important to the successful development of the IDEA Strategy: creating space to acknowledge differences and navigate conflict, recognising the value of skilled facilitation, and ensuring visible and sustained leadership commitment.

### Lesson 1: Creating Space to Acknowledge Differences and Navigate Conflict

The first lesson is to ensure processes exist for acknowledging differences across the group, with the aim of preventing and sorting out conflicts. Co-creation and collaborative strategy development requires fostering psychological safety, naming and identifying power dynamics, and agreeing on shared rules of engagement [19, 20]. Shortly after embarking on this project, we realised that while all Project Team members value IDEA, collective buy-in was not guaranteed. Individual team members’ experiences with IDEA and the values and priorities they held influenced their decision to participate and desire to create something meaningful, especially in a context where IDEA-related outputs do not always result in expected change [55]. At times, this resulted in a difference of opinion on how to proceed with strategy development. Establishing the project charter at the outset and clearly defining the consensus-based approach to decision making was critical for naming power as an essential factor, creating space for people to share their opinions, values, beliefs, and providing a reference point for team commitment and accountability.

### Lesson 2: the Importance of Skilled Facilitation

Our second lesson is not to underestimate the importance of facilitation. Co-creation requires significant skills and expertise to manage the complexity and conflict that can emerge in group processes [56]. Recognising that HDRN Canada members had the technical expertise in population health research and conceptual expertise in IDEA, we ultimately decided hiring a skilled facilitator who could navigate the challenges of bringing people together across an extensive, geographically dispersed network would yield a better return on investment. Noting that the quality of facilitation has a profound impact on an initiative’s success [57], it was important that a key selection criterion would be to have a clear process that would address concerns about how much time and resources would be needed to complete the strategy. The facilitation kept the focus on possibilities rather than barriers. This was not just for the pragmatic reason of ensuring we could complete the strategy activities, but also recognised that the complexity of IDEA itself could end up being a significant barrier to adopting the IDEA strategy [58]. Relying on an expert to help balance power among participants and enable different voices to influence discussions and decisions is necessary for fostering inclusion and shared ownership needed for co-creation [20].

### Lesson 3: Leadership Commitment Creates the Conditions for Change

Our final and perhaps most fundamental lesson was that visible and sustained leadership commitment creates the conditions necessary for meaningful organisational change. Despite having identified a clear imperative for HDRN Canada to focus efforts on embedding IDEA within and across the network, efforts to develop and implement the IDEA strategy would not be possible without support from the Executive Team and Board of Directors. Implementation and organisational change research consistently reports that visible and sustained executive sponsorship is pivotal to success in complex, equity-oriented initiatives [59–61]. By having executive and director-level participation in strategy sessions, roundtable discussions, and town hall presentations, we were not only able to signal that IDEA was an organisational priority but also secure the necessary resources for strategy development and implementation. Leadership participation also modelled an openness to the change and transformation needed to achieve the IDEA Strategy’s objectives and goals. Ultimately, executive sponsorship, engagement, and visibility throughout the strategy development process provided the authority, resources, trust, and shared ownership necessary to translate the IDEA strategy from a concept into something meaningful and actionable for HDRN Canada.

## Conclusion

HDRN Canada’s IDEA Strategy demonstrates how a distributed research network can respond to the growing imperative for Inclusion, Diversity, Equity, and Accessibility (IDEA) by developing a structured and contextually relevant strategic framework. By describing the process used to co-create the strategy, this paper addresses a gap in the literature regarding how organisations can translate IDEA principles into priorities and actions tailored to complex research environments.

The strategy was developed through an organisation-wide process that included preparatory work to assess readiness and identify priorities, broad engagement across the network, iterative feedback, and visible executive sponsorship. Together, these activities helped ensure that IDEA is not regarded as an add-on, but as a core consideration within HDRN Canada’s governance, operations, and research infrastructure. The resulting strategy is organised around four interrelated priorities: Learning and Unlearning, Facilitating IDEA in Research, Cultivating Trust and Reciprocity, and Providing Leadership and Advocacy, which are intended to address structural and epistemic harms embedded within population health research and to guide ongoing organisational change.

Since the strategy was approved, implementation has been underway across all four priorities. Initial and ongoing activities include developing IDEA core competencies, creating briefing notes to support the integration of IDEA into administrative data research infrastructure, establishing processes to consider IDEA and public engagement in internal projects and initiatives, and publicly sharing the strategy to support broader dialogue within the population health research community.

Rather than evaluating implementation outcomes, this paper focuses on the participatory and consensus-based approach used to co-create the strategy. In doing so, it offers a replicable model for organisations seeking to embed IDEA within the technical and cultural architecture of population health research. As implementation continues, future publications will describe the activities, outcomes, and lessons arising from this work. HDRN Canada’s experience serves as both a practical roadmap and an invitation to reimagine a population health research ecosystem that is more just, inclusive, and equitable.

## Data Availability

The data associated with this project are not available for public dissemination.
